# Associations of Maternal Pre‐Pregnancy BMI With Frontostriatal Connectivity in Young Children

**DOI:** 10.1111/ijpo.70101

**Published:** 2026-03-29

**Authors:** Abdulmumin Ibrahim, Liuyi Chen, Elena Jansen, Muriel M. K. Bruchhage, Jennifer Beauchemin, Angelica Owen, Rosa Cano Lorente, Fatoumata Barry, RESONANCE CONSORTIUM, D. Koinis‐Mitchell, Viren D'Sa, Sean C. L. Deoni, Susan Carnell

**Affiliations:** ^1^ Division of Child and Adolescent Psychiatry, Department of Psychiatry and Behavioral Sciences Johns Hopkins University School of Medicine Baltimore Maryland USA; ^2^ Department of Psychology, Institute of Social Sciences University of Stavanger Stavanger Norway; ^3^ Stavanger Medical Imaging Laboratory, Department of Radiology University Hospital Stavanger Norway; ^4^ Advanced Baby Imaging Lab Rhode Island Hospital Providence Rhode Island USA; ^5^ Department of Pediatrics, Warren Alpert Medical School Brown University Providence Rhode Island USA; ^6^ Maternal, Newborn and Child Health Discovery & Tools Bill & Melinda Gates Foundation Seattle Washington USA

**Keywords:** eating behaviour, executive function, frontostriatal networks, maternal obesity, neurodevelopment, overweight

## Abstract

**Background:**

Maternal obesity during pregnancy has previously been associated with increased body weight and altered brain structure and function in offspring. However, few studies have included young children or specifically examined effects on functional connectivity of frontostriatal networks undergirding functions implicated in appetite and body weight, such as reward processing and inhibitory control.

**Methods:**

We analysed resting state functional Magnetic Resonance Imaging (rsfMRI) data for 83 children (ages 2–11 years) from the RESONANCE cohort who were born to mothers ranging in BMI as assessed pre‐pregnancy (BMI: mean ± SD = 26.8 ± 7.8, range = 18.3–50.1). We examined associations between maternal BMI and child functional connectivity of the nucleus accumbens (NAcc) with three regions in the frontal cortex.

**Results:**

Analyses controlling for maternal education and for child sex, age and current BMIz demonstrated that higher maternal pre‐pregnancy BMI was associated with stronger positive connectivity of NAcc with rostral prefrontal cortex and frontal pole. Stratified sensitivity analyses demonstrated that child effects were driven by weight variation among mothers with higher weights rather than representing an effect of lower maternal weight.

**Conclusion:**

Our analysis found that increased BMI during pregnancy was associated with stronger positive frontostriatal connectivity in young children. Prospective studies incorporating gold‐standard measures of maternal and child adiposity and metabolic health as well as behavioural and cognitive measures are needed to understand implications and inform future interventions.

## 1 Introduction

Maternal obesity during pregnancy has implications for offspring health. Children born to mothers with obesity are at elevated risk for increased body weight and obesity as well as for poorer neurodevelopmental outcomes, with emerging evidence suggesting that maternal obesity can disrupt offspring neurodevelopment, leading to alterations in brain structure and function, as well as increased risk for poorer cognitive outcomes [1, 2, 3]. Several factors are implicated in the intergenerational transmission of obesity risk, such as the upsurge of pro‐inflammatory cytokines in the intrauterine environment, disrupting foetal brain development via increased oxidative stress, and changes in hormonal regulation [3, 4]. Specifically, maternal obesity and a high‐fat diet can affect dopamine and opioid signalling in offspring, influencing food preferences and reward related behaviours [5].

Preclinical and clinical Magnetic Resonance Imaging (MRI) studies in adults and children together implicate a network of distributed brain regions, including frontostriatal circuits involved in self‐regulatory processes and reward mechanisms that drive eating behaviour and thereby body weight [6, 7]. The frontostriatal circuitry, encompassing the nucleus accumbens (NAcc) and regions in the prefrontal cortex (PFC), plays a critical role in regulating appetite, reward processing, and inhibitory control over food intake. Disruptions in the development and function of this circuitry may contribute to dysregulated eating behaviours and increased vulnerability to obesity [8, 9]. In particular, the involvement of the NAcc is underscored by preclinical studies of diet‐induced obesity, which show that microstructural changes consistent with a neuroinflammatory response influence food‐seeking behaviour [10, 11]. Evidence from both human and animal studies also implicates involvement of frontal brain regions [12, 13, 14].

Although basic science and neurodevelopmental studies support that maternal obesity impacts the brain in offspring, and basic science and clinical MRI studies have implicated frontostriatal circuits in individual variation in appetite and body weight, relatively few human studies have directly tested whether maternal obesity affects frontostriatal brain function, which is possible by using functional MRI data acquired in a resting state functional Magnetic Resonance Imaging (rsfMRI). rsfMRI offers a non‐invasive approach to investigate stable functional organisation of the brain in young children. By measuring spontaneous brain activity during a resting state, rsfMRI provides insights into the intrinsic connectivity between brain regions [15] and can therefore identify potential disruptions in functioning of neural circuitry associated with obesity risk.

A small body of literature has applied varying statistical methods to rsfMRI data in young infants to demonstrate associations of pre‐pregnancy maternal weight with alterations in brain function [16, 17, 18, 19, 20], revealing some evidence of altered connectivity of frontal regions with other brain regions. In parallel, food cue fMRI studies examining associations with current maternal weight have demonstrated altered engagement of frontocingulate regions in middle childhood [18] and adolescence [21]. However, since these samples did not span early childhood, and brain development is dynamic and rapid in early life [22], it is unclear whether early‐observed alterations persist beyond infancy, or whether later brain outcomes reflect effects of maternal weight or rather postnatal environmental factors (e.g., dietary exposures) or correlates of growth (e.g., metabolic or inflammatory sequelae). One study of 4–6‐year‐olds assessed functional connectivity while viewing a movie and observed weaker functional connectivity of right inferior frontal gyrus with paracingulate gyrus among children of mothers with pre‐pregnancy maternal overweight or obesity [18]. However we are not aware of any study that has examined associations of pre‐pregnancy maternal weight with resting state connectivity in children beyond infancy but below 7 years, or focused specifically on function within frontostriatal regions implicated in appetite.

Early childhood is a critical period for PFC development, marked by rapid neural growth and heightened sensitivity to environmental influences [23]. Furthermore, early childhood eating behaviours and neural circuit development shape lifelong dietary habits and obesity risk [24, 25]. Maternal obesity during early development can program long‐lasting changes in offspring brain circuits regulating appetite and metabolism [26]. While early childhood is a critical period, investigating children through middle childhood is also critical because it allows examination of how early disruptions related to maternal obesity may persist over time, and of long‐term consequences of early environmental exposures on brain development [27, 28].

In summary, while a handful of previous fMRI studies have investigated the impact of maternal weight on brain function in early life, significant gaps remain. First, very few studies have examined functional connectivity using rsfMRI, which provides important insights into communication between brain regions. Second, most of the relevant extant rsfMRI studies have focused on infancy, leaving a critical gap in our understanding of how maternal weight impacts brain development in young children. Third, limited studies have focused on frontostriatal circuits, which are critical in orchestrating the balance between food reward and self‐control.

To extend the above literature we therefore sought to probe potential impacts of maternal obesity during pregnancy on the functioning of frontostriatal brain regions implicated in appetite and obesity among young children.

We leveraged rsfMRI data from RESONANCE, a longitudinal multimodal study of early brain development from infancy through childhood. Based on the above reviewed evidence for the role of NAcc and frontal cortex in appetite and obesity risk in children, and previous RESONANCE analyses of a larger dataset demonstrating structural alterations in NAcc in association with genetic obesity risk [29], we examined connectivity of NAcc with three regions in the frontal cortex that have been implicated in appetite and weight variation [30, 31, 32, 33] in a sample of children aged 2–11 years, and investigated the association between frontostriatal functional connectivity and maternal pre‐pregnancy BMI. We hypothesised that higher maternal pre‐pregnancy BMI would be associated with altered frontostriatal connectivity, with potential implications for appetite, behaviour/cognition and body weight.

## 2 Methods

### 2.1 Participants

For the current analysis, cross‐sectional data were drawn from the longitudinal RESONANCE cohort, which forms part of the NIH‐funded ECHO program (http://echochildren.org) and includes multimodal neuroimaging [34, 35] enriched with measures of factors relevant to obesity risk [25]. Recruitment (e.g., flyers, social media, in person events) of participants took place during pregnancy or when children were aged between 0 and 5 years [36]. Paediatric participants were followed with biannual visits up to the age of 2 years, and annual visits beyond that. Children with known risk factors for learning and/or psychiatric disorders were excluded (e.g., birth prior to 32 weeks gestation or birth weight < 1500 g, non‐singleton or complicated pregnancy, neurological trauma in child, psychiatric history in parent or sibling) [34]. Written consent was obtained from parents or legal guardians in accordance with ethics approval from the host institution's Institutional Review Board. For the current analysis we included only children ≥ 2 year who had a complete, high quality anatomical rsfMRI dataset, and had data available on maternal pre‐pregnancy BMI and education, and child age, sex, race and ethnicity.

### 2.2 Measures

Maternal pre‐pregnancy BMI was based on self‐reported weight and height. Participants could enrol at different stages into RESONANCE, and pre‐pregnancy weight and height were assessed as soon as possible upon entry into the study, that is retrospectively during infancy or early childhood. Pre‐pregnancy BMI was used to form four weight categories: < 18 = underweight, 18.5–24.9 = healthy weight, 25.0–29.9 = overweight, ≥ 30.0 = obesity. The following information was additionally collected and used either for descriptive purposes or for inclusion as covariates into adjusted models: child age, sex, race and ethnicity; maternal education level. Child weight and height were directly measured by study staff during assessments and child BMI *z*‐scores were calculated using the WHO Anthro version 3.2.2., allowing a similar metric to be applied across the whole RESONANCE sample, which includes infants. Further, to promote comparability with previous research conducted in the US, child weight groups were generated based on CDC reference data [37].

### 2.3 Imaging Acquisition and Processing

All MRI data were acquired on a 3T Siemens Trio scanner with a 12‐channel head RF array. rsfMRI data were acquired using the following parameters (for more details, see [34]): TE = 34 ms, TR = 2.5 s, flip angle = 80°, field of view = 24 × 24 cm^2^, imaging matrix = 80 × 80, 32 interleaved 3.6 mm slices (for a voxel resolution: 3 × 3 × 3.6 mm^3^), BW = 751 Hz/pixel and GRAPPA acceleration factor of 2. We acquired 164 volumes for a total acquisition time of approximately 7 min. As described elsewhere [34], to achieve successful scanning without sedation, scans for younger children (< 4.5 year) were scheduled around the child's usual nap time. Once asleep, the child was transferred to the scanner bed. To maintain sleep state, we used noise‐absorbing foam inside the bore, headphones, and custom MRI pulse sequences with reduced gradient slew rates to reduce noise levels. Children were positioned in an immobiliser to keep them still and monitored visually by research staff at the scanner bed as well as outside the scanner using an infra‐red camera. Physiological monitoring via a pulse‐oximeter was also used to identify child distress as well as to time scans to periods of deep sleep.

T1‐weighted magnetisation‐prepared rapid acquisition gradient echo anatomical data were acquired with an isotropic voxel volume of 1.2 × 1.2 × 1.2 mm^3^, resampled to 0.9 × 0.9 × 0.9 mm^3^ Sequence specific parameters were: TE = 6.9 ms; TR = 16 ms; inversion preparation time = 950 ms; flip angle = 15°; BW = 450 Hz/Pixel. The acquisition matrix and field of view were varied according to child head size in order to maintain a constant voxel volume and spatial resolution across all ages [38]. Image registration followed a multi‐step registration procedure using high resolution T1‐weighted images collected in each dataset. These templates were used as initial targets for nonlinear registration from ‘native space’ acquired high resolution T1‐weighted images. A pre‐calculated non‐linear warp from these age‐specific templates to a common/standard paediatric space were then used to move all images with all transformations performed in a single interpolation step. The Advanced Normalisation Tools (ANTs) package [39] was used for non‐linear registration and FSL FLIRT [40] for linear and affine registrations.

To obtain functional connectivity values, the rsfMRI data were first pre‐processed (including realignment, centring, motion correction and scrubbing) using the CONN‐fMRI toolbox for SPM [41] on MATLAB and registered to our child study template using the FSL FLIRT [40] and ANTs [39]. Region of Interest (ROI)‐to‐Region of Interest (ROI) connectivity analyses were then performed, computing the correlation of spontaneous BOLD activity between network regions. A total of 32 cortical and subcortical anatomical bilateral ROIs based on the network atlas described in [41] were used in the network analysis and warped to our child study template using the ANTs package [39] for non‐linear registration and FSL FLIRT [40] for linear and affine registrations. A reference of the network distribution in the child template can be found in the Supporting Information (Figure S1). Using CompCor [42], the effect of nuisance covariates including BOLD signal fluctuations from CSF, white matter and their derivatives, as well as the realignment parameter noises were reduced. Data were simultaneously band‐pass filtered (0.008 < *f* < 0.09 HZ) and global signal regression was applied, including derivatives. Finally, we scrubbed volumes if there was significant motion during data acquisition (i.e., DVARS > 5 or framewise displacement, FD were > 0.5) [43]. Mean participant head motion as calculated by average FD was FD = 0.28 (standard deviation = 0.23), which represents moderate but acceptable levels in the context of motion scrubbing as previously specified [44].

## 3 Statistical Analysis

We conducted statistical analyses using the R statistical software [45]. To compare sample characteristics between children born to mothers with healthy‐weight versus overweight/obesity pre‐pregnancy, we used Student's *t*‐tests for continuous variables and Chi‐square tests for categorical variables.

Our primary analysis used linear regression to investigate associations of maternal pre‐pregnancy BMI with child resting state functional connectivity metrics across all participants. For the current analyses focusing on frontostriatal connectivity we analysed only metrics describing functional connectivity of left and right NAcc with left and right rostral PFC, lateral PFC and frontal pole. Extracting connectivity metrics for all possible bilateral combinations resulted in 12 metrics in total. Prior to the ROI‐to‐ROI analysis, we pre‐processed the data using a network atlas of 32 cortical and subcortical ROIs from [41]. We focused on the NAcc, rostral PFC, lateral PFC and frontal pole because these regions are key components of the frontostriatal circuitry involved in reward processing, inhibitory control and decision‐making related to food intake, and previous research has implicated these regions in the pathophysiology of obesity and related eating disorders [30, 31, 32, 33]. Before proceeding to the regression analyses, we first ran Pearson correlations to determine the most salient covariates (Table S1). Given that several potential confounders or variables were related to connectivity metrics at *p* < 0.05 and *p* < 0.1, we included all variables as covariates, across all models. Confounding variables included were child age, sex, BMI *z*‐score and maternal education level. For all analyses, we used *p* < 0.05 for hypothesis testing and further applied the false discovery rate (FDR) method to adjust for multiple comparisons for the main analyses.

In addition, for clinical relevance we conducted two sets of sensitivity analyses. First, to test whether results were driven by variation at the upper or lower end of the maternal adiposity range, we repeated linear regression analyses stratifying by maternal weight (healthy‐weight, overweight and obesity). Second, to explore whether results were driven by older or younger children or by sex, we repeated linear regression analyses stratifying by child age (≤ 5 years, > 5 years) and by sex (female vs. male). Child age was divided into two categories: 5 years or younger and older than 5 years, based on evidence suggesting that brain development undergoes significant changes around this age [46]. These age categories approximately correspond with pre‐school versus school age. Child sex was coded as boy and girl. We used the same linear regression models as for the main analyses to examine the relationships between maternal pre‐pregnancy BMI and child brain connectivity within each subgroup.

## 4 Results

### 4.1 Sample Characteristics

Characteristics of our analysis sample are described in Table 1. We analysed processed rsfMRI data for 83 children (mean age = 5.0 ± 2.1, range = 2.0–9.8; mean BMIz = 0.2 ± 1.1, range = −1.9–3.4; 39 female, 44 male) with complete data on key variables and relevant confounders of whom 36 were born to mothers with overweight/obesity as assessed pre‐pregnancy, and 47 were born to mothers with healthy‐weight as assessed pre‐pregnancy (mean maternal BMI = 26.8 ± 7.8). Children born to mothers in the overweight/obesity group were slightly younger than those born to healthy mothers. About a third of mothers had university or college education, with no differences by maternal weight category. Regarding gestational weight gain (GWG), absolute GWG was comparable across all BMI categories (*p* > 0.05). However Chi‐square analyses revealed that the proportion of mothers with excessive GWG based on Institute of Medicine (IOM) recommendations differed by group such that more mothers in the overweight and obesity groups had excessive weight gain compared to the healthy‐weight group.

**TABLE 1:** Sample characteristics.

	All (*n* = 83)	Maternal pre‐pregnancy BMI group (*n* = 83)			*p*
		Healthy‐weight (*n* = 47)	Overweight (*n* = 13)	Obesity (*n* = 23)	
Maternal BMI	28.2 ± 9.3	21.5 ± 1.8	27.1 ± 1.3	37.6 ± 6.1	**0.0001**
Mother's highest level of education					
No university/college	28 (33.7%)	17 (36.2%)	5 (38.5%)	6 (26.1%)	0.65
University/college	55 (66.3%)	30 (63.8%)	8 (61.5%)	17 (73.9%)	
Child age (years)	5.0 ± 2.1	5.6 ± 2.3	3.8 ± 1.4	4.8 ± 1.9	**0.001**
Child sex					
Female	39 (47.0%)	23 (48.9%)	7 (53.8%)	9 (39.1%)	0.64
Male	44 (53.0%)	24 (51.1%)	6 (56.2%)	14 (60.9%)	
Child BMIz^a^	0.2 ± 1.1	0.04 ± 1.2	0.09 ± 0.9	0.5 ± 1.0	0.93
Child weight category^a^					
Healthy weight	48 (66.7%)	30 (62.5%)	10 (45.4%)	0	0.17
Overweight	2 (2.8%)	12 (25.0%)	8 (36.4%)	2 (100%)	
Obesity	22 (30.5%)	6 (12.5%)	4 (18.2%)	0	
Child ethnicity					
Hispanic/Latino	17 (19.3%)	8 (17.1%)	4 (30.8%)	5 (21.7%)	0.72
Non‐Latino/Hispanic	66 (79.5%)	38 (80.8%)	9 (69.2%)	19 (78.3%)	
Unknown	1 (1.2%)	1 (2.1%)	0	0	
Child race					
Black/African American	6 (7.2%)	1 (2.1%)	2 (15.4%)	1 (4.3%)	**0.003**
White	68 (81.9%)	37 (78.8%)	8 (61.5%)	19 (82.7%)	
Mixed	6 (7.2%)	7 (14.9%)	1 (7.7%)	3 (13.0%)	
Others	3 (3.6%)	2 (4.2%)	2 (154%)	0	
Gestational weight gain (GWG) (kg)^b^	11.8 ± 5.3	11.3 ± 5.7	11.88 ± 4.6	13.0 ± 5.1	0.61
Adequate	25 (35.7%)	19 (51.4%)	1 (7.8%)	5 (25.0%)	0.0001
Inadequate	17 (24.3%)	14 (37.8%)	0	3 (15.0%)	
Excessive	28 (40.0%)	4 (10.8%)	12 (92.2%)	12 (60.0%)	

*Note:* Values are mean ± SD or *n* (%). One‐way ANOVA was used to test differences by maternal pre‐pregnancy BMI group for continuous variables and Chi‐square tests for categorical variables. Bold text indicates statistical significance. IOM‐GWG Recommendation: Healthy weight = 11–16 kg; Overweight = 7–11 kg; Obesity = 5–9 kg; *p* < 0.05.

^a^ 11 missing.

^b^ 13 missing.

**FIGURE 1:**
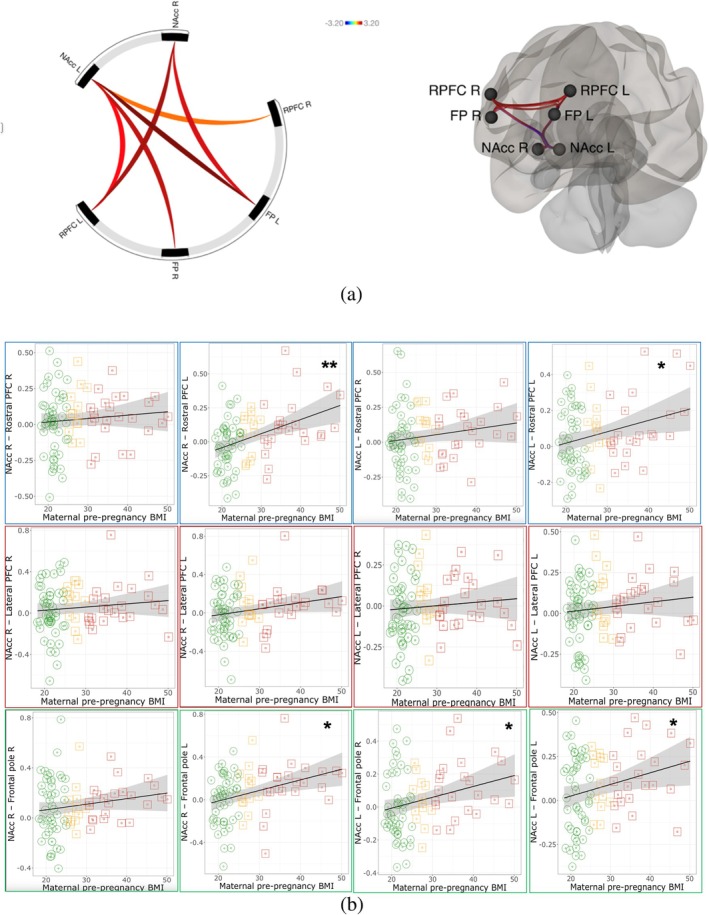
(a) Regions showing differential connectivity in relation to maternal pre‐pregnancy BMI. The colour of each edge corresponds to the *T* statistic from the second‐level general linear model in CONN controlling for maternal education, and child age, sex, and BMIz. Warmer colours (red to orange) indicate stronger connectivity with higher maternal pre‐pregnancy BMI. The colour scale represents *T* values ranging from −3.20 (blue, not shown) to +3.20 (red). (b) Plots showing associations of maternal pre‐pregnancy BMI with child functional connectivity outcomes. Figures depict all relationships before control for confounders. * Indicates *p* < 0.05 and ** Indicates pFDR < 0.05 in main analyses with control for potential confounders. FP, frontal pole; L, left; NAcc, nucleus accumbens; PFC, prefrontal cortex; R, right; RPFC, rostral prefrontal cortex.

Results of models testing relationships of maternal pre‐pregnancy BMI with functional connectivity metrics across all children (Figure 1), adjusting for confounders, are described in Table 2 (summary of regression results across whole sample) and Table S2 (full regression results across whole sample including effects of covariates). Figure 1b depicts regression results across whole sample before control for confounders.

**TABLE 2:** Regression coefficients for effect of maternal pre‐pregnancy BMI on child functional connectivity metrics (*n* = 83).

Connectivity with NAcc	*β* (SE)	*p*	pFDR
Rostral PFC			
NAcc R—Rostral PFC R	0.004865 (0.003745)	0.200	0.3000
NAcc R—Rostral PFC L	0.009581 (0.003682)	**0.0119**	**0.0476**
NAcc L—Rostral PFC R	0.000789 (0.000381)	**0.0429**	0.0858
NAcc L—Rostral PFC L	0.008655 (0.003204)	**0.00921**	0.1105
Lateral PFC			
NAcc R—Lateral PFC R	0.005633 (0.004398)	0.2057	0.2743
NAcc R—Lateral PFC L	0.004279 (0.004351)	0.3298	0.3598
NAcc L—Lateral PFC R	0.003434 (0.003525)	0.3343	0.3343
NAcc L—Lateral PFC L	0.003495 (0.003327)	0.2981	0.3577
Frontal pole			
NAcc R—Frontal pole R	0.007537 (0.003874)	0.0569	0.0975
NAcc R—Frontal pole L	0.009311 (0.004452)	**0.0412**	0.0988
NAcc L—Frontal pole R	0.009689 (0.003617)	**0.00978**	0.0587
NAcc L—Frontal pole L	0.007822 (0.003382)	**0.02458**	0.0737

*Note:* Models adjust for maternal education, and child age, sex and child BMIz. Bold indicates statistical significance; *p* < 0.05.

Abbreviations: L = left; NAcc = nucleus accumbens; PFC = prefrontal cortex; pFDR = *p* value following FDR correction for multiple comparisons; R = right.

FDR‐corrected regression models demonstrated that higher maternal BMI was associated with stronger positive functional connectivity between right NAcc and left rostral PFC (*p* = 0.012, pFDR = 0.047). Models without FDR correction additionally found that higher maternal BMI was associated with stronger positive functional connectivity of left NAcc with bilateral frontal pole (right *p* = 0.009, left *p* = 0.02) and bilateral rostral PFC (right *p* = 0.04, left *p* = 0.009), and of right NAcc with left frontal pole (*p* = 0.04) and left rostral PFC (0.01). We assessed multicollinearity in our regression models by calculating Variance Inflation Factors (VIFs). The VIF for maternal pre‐pregnancy BMI was 1.27, and the VIF for child BMI *z*‐score was 1.11. These values suggest that multicollinearity was not a major concern in our models [47].

Effects of covariates within the full regression results are important to consider when interpreting the results Table S2. Our results indicated that sex was most robustly associated with frontostriatal functional connectivity, with males demonstrating stronger connectivity on multiple metrics. In contrast, associations for age and maternal education were more localised. Age was positively associated with connectivity specifically for right NAcc to right lateral PFC but showed no significant associations with other metrics (*ps* > 0.075). Maternal education was negatively associated with connectivity of right NAcc to left lateral PFC (*β* = −0.0624, *p* = 0.0483), with no significant associations for other metrics. Child BMIz scores did not demonstrate any significant associations with functional connectivity across any of the examined metrics in the full regression models.

Table S3 describes the results of additional regression analyses stratified by maternal BMI status. Despite the small sub‐group size, 4/6 of the whole sample results were replicated when confining the analysis just to children born to mothers with obesity pre‐pregnancy, supporting a dose response effect of adiposity rather than variation in body mass among the leaner mothers. Further, the left NAcc‐left rostral PFC functional connectivity finding in the whole sample was significant within both the maternal overweight and the maternal obesity sub‐groups, with *β* values being higher for both of these sub‐group analyses than for the whole sample result including children born to mothers with healthy‐weight pre‐pregnancy.

Tables S4 and S5 describe results of additional regression models stratified by child sex and child age group respectively. Results suggested that whole sample results were substantially driven by males, with the exception of the right NAcc‐left rostral PFC finding, which reached statistical significance in both males and females. Age‐stratified results suggested that 4/6 of the whole sample results were substantially driven by older (> 5 year) children.

## 5 Discussion

We investigated the effects of maternal pre‐pregnancy BMI variation on resting‐state functional connectivity in children from 2 to 11 years, focusing on the NAcc and its interactions with regions in PFC. The NAcc is critically involved in reward processing, motivation and reinforcement learning including in relation to food reward, with frontostriatal circuits subserving functions including self‐regulation and decision‐making in relation to food [48, 49], making NAcc‐PFC connectivity a candidate substrate for intergenerational transmission of obesity risk. Our analyses of the sample as a whole revealed a pattern of stronger positive ipsilateral and contralateral connectivity of NAcc with both rostral PFC and frontal pole, among children born to mothers with higher pre‐pregnancy BMI, with the increased connectivity of right NAcc with left rostral PFC withstanding correction for multiple comparisons. Analyses testing differences by maternal pre‐pregnancy BMI group and associations with maternal pre‐pregnancy BMI within weight groups confirmed these findings were driven by maternal weight variation at the upper end of the distribution, consistent with a dose response effect of adiposity. Our most robust finding was the association of maternal pre‐pregnancy BMI with stronger positive connectivity of right NAcc to left rostral PFC. Further, this association was strongest among children born to mothers with obesity (as opposed to overweight or healthy‐weight), suggesting that this functional pathway is affected by the degree of excess adiposity rather than being driven by variation in body mass or adiposity at the lower end of the weight distribution.

Our analysis of the relationships of brain connectivity metrics with maternal education, child age and sex, and BMIz did not reveal pervasive evidence for associations across all metrics; however, these variables were nonetheless included in our models to ensure that our findings were not unduly driven by these variables. Sensitivity analyses stratified by child sex and age suggested that the findings were substantially driven by males and children over 5 years of age. Moderation analyses within larger datasets could probe these findings to establish whether effects of pre‐pregnancy maternal BMI on frontostriatal connectivity are meaningfully greater in boys and in older children. We note, however, that differences in functional connectivity with age could also be driven in part by whether rsfMRI was acquired while sleeping or while awake. In our sample, children under 5 years were more likely to have data acquired while napping, which introduces a potential confound common to many developmental neuroimaging studies [50] that must be considered when interpreting age effects.

Our results align with previous studies suggesting that maternal obesity can influence offspring neurodevelopment and cognitive outcomes [2, 51, 52], and build on previous studies linking variation in maternal pre‐pregnancy BMI to resting state functional connectivity alterations in infants. For example, Rajasilta et al. [16] found in 21 healthy neonates that higher maternal pre‐pregnancy BMI was associated with stronger positive connectivity of the left superior frontal gyrus with the ventral striatum, while Salzwedel et al. [19] in 38 full‐term neonates observed an overall pattern of heightened functional connectivity across brain regions in association with higher maternal adiposity quantified via either BMI or body fat mass percentage. Our findings in 2–11 year olds suggest that the effects of maternal BMI on functional connectivity may persist beyond infancy and into early childhood, and specifically demonstrate the effects of maternal BMI on connectivity between NAcc and frontal cortex regions, which may be of particular relevance to appetite and obesity.

The observed stronger connectivity between the NAcc and PFC regions in children of mothers with higher pre‐pregnancy BMI could reflect several underlying neurobiological mechanisms. One possibility is that maternal obesity‐related factors, such as altered nutrient availability or increased inflammation during pregnancy [4, 53] disrupt typical synaptic pruning processes in the developing brain. This could lead to an excess of connections within the frontostriatal circuitry, resulting in a less efficient but more strongly connected network. Alternatively, the hyperconnectivity may represent a compensatory response to early disruptions in reward circuitry. In this scenario, the brain may be attempting to overcome impaired dopamine signalling or reduced reward sensitivity [5] by strengthening connections between the NAcc and PFC. We note this is similar to other phenomena in developmental neuroscience where early enlargement/hyperactivity of the NAcc results in excess signal outputs and perhaps the receipt of overly complex inputs, leading to dysregulation of brain‐behaviour relationships, which could include eating behaviours and perhaps central modulation of peripheral metabolism [54]. Consequently, the circuits overcompensate to normalise the firing and size of the structure later in development, resulting in over‐pruning (neuronal cell death and process retraction) to manage the exuberant complexity [55]. Regardless of the underlying mechanism, this altered frontostriatal connectivity could have significant implications for the development of eating behaviours and obesity risk. Increased connectivity between the NAcc and PFC could potentially lead to heightened reward responses toward palatable foods, reduced inhibitory control over food intake, and increased impulsivity in food‐related decision‐making [8, 9]. Longitudinal studies are needed to determine whether this early hyperconnectivity is robust across offspring of mothers with higher weight, and whether it predicts future weight gain and metabolic dysfunction, as well as the degree to which the relationship between maternal pre‐pregnancy BMI and offspring brain connectivity is mediated by the child's own adiposity or metabolic profile, which in turn influences brain development.

The current study lacked integration of behavioural and cognitive measures to fully understand implications of observed frontostriatal connectivity differences for eating behaviour and associated obesity risk. However we speculate that the differences we observed could reflect an impact of maternal obesity on the development of circuits undergirding reward processing and executive function, with impacts on child eating behaviour. In support, previous studies have demonstrated associations of maternal pre‐pregnancy or current obesity with heightened appetite in childhood [56, 57] and fMRI studies by ourselves and others have found associations of maternal pre‐pregnancy or current obesity with altered engagement of frontocingulate regions in middle childhood [18] and adolescence [21]. Alterations in functional connectivity between the NAcc and prefrontal regions may therefore reflect individual differences in the balance between reward processing and executive control, with ultimate effects on eating behaviour. Previous studies have shown that children born to mothers with obesity show poorer executive functioning and increased impulsivity, functions subserved by the PFC [58, 59]. Our findings extend these findings by suggesting that alterations in PFC functioning may be reflected in frontostriatal connectivity patterns as early as 2 years of age.

We note that the bilateral nature of our findings, which included ipsilateral and contralateral connections of left and right hemisphere structures (though not all findings withstood FDR correction), suggests that the impact of maternal pre‐pregnancy BMI on child brain development is relatively widespread. This is consistent with prenatal exposure to maternal obesity potentially having global effects on foetal brain development through mechanisms including inflammation, altered nutrient transfer, or hormonal imbalances [60].

Relationships of child adiposity with frontostriatal connectivity were not the primary focus of this study. However, we found that higher child BMIz was associated with significantly greater connectivity of left NAcc with both left and right lateral PFC, a critical region for executive function, possibly reflecting effects of obesity risk factors on neurobehavioural mechanisms driving weight gain, rather than effects of maternal adiposity and associated hormonal milieu on the brain. This is in striking opposition to our findings for maternal pre‐pregnancy BMI, which revealed no differences for lateral PFC, and provides further confirmation that the maternal pre‐pregnancy BMI effects we observed are attributable to maternal BMI rather than child BMI. Reduced PFC volumes and altered connectivity with higher child weight, including evidence of heightened connectivity between NAcc and frontoparietal regions [61] have been consistently observed in large cohorts such as ABCD [13, 62] and our results extend these by additionally demonstrating that altered frontostriatal connectivity with higher BMI is also evident earlier in development.

We acknowledge that changes in maternal BMI during pregnancy are likely important for offspring brain development, potentially attenuating or exacerbating the effects of pre‐pregnancy BMI. GWG is a critical factor in pregnancy, influencing both maternal and infant health outcomes. Both the amount and pattern of weight gained during pregnancy, as well as a woman's BMI before conception, are strongly associated with risks for complications such as gestational diabetes, hypertensive disorders, caesarean delivery, and abnormal birth weights [63]. Changes in maternal BMI during pregnancy, independent of pre‐pregnancy BMI, may influence offspring neurodevelopment. For example, women who developed overweight or obesity during pregnancy may expose their offspring to different intrauterine conditions compared to women who maintained a stable, healthy weight, and children of women who limited their GWG during pregnancy may have demonstrated different neurodevelopmental effects compared to children of mothers who had excess GWG during pregnancy. We did not see an absolute difference in GWG between maternal BMI groups in the current study. However, the prevalence of excess weight gain relative to clinical guidelines was significantly higher in the overweight and obesity groups. Mothers in the obesity category gained an average of 13.0 ± 5.1 kg, exceeding the IOM recommended maximum (9 kg) by approximately 4.0 kg. In contrast, mothers in the normal weight category (11.3 ± 5.7 kg) remained within their recommended 11–16 kg range. The excess GWG in mothers with obesity—representing a significant metabolic deviation from clinical target—may create an intrauterine environment that influences the development of reward‐regulatory circuits [64], contributing to our observations of higher frontostriatal connectivity in children born to mothers with higher weight. Maternal BMI changes during pregnancy can affect offspring development through multiple interconnected mechanisms, including changes in maternal glucose levels [65], changes in maternal inflammation, oxidative stress, changes in hormone levels [3, 4] and changes in nutrient availability to the foetus [66].

Study limitations include the use of retrospectively self‐reported height and weight to define maternal pre‐pregnancy BMI, which is subject to recall bias and inaccuracies. However, maternal self‐report of pre‐pregnancy weight has been demonstrated to have relatively high accuracy and there is relatively close correspondence between BMI groups as estimated from both measured and self‐reported weight [67]. We were unable to account for other factors such as maternal diet, physical activity, and stress levels during pregnancy. Neither did we address the potential contribution of current maternal obesity which is likely highly correlated with pre‐pregnancy weight and might act as a proxy for environmental factors that could impact children's frontostriatal functioning in children. Future work explicitly measuring and evaluating such factors is warranted. Future studies should also collect data on maternal BMI as well as diet at multiple time points during pregnancy to better characterise the dynamic changes in maternal weight status and dietary intake and their potential impact on offspring brain development.

Another important methodological limitation is not including sleep or wakefulness as a covariate in our model, which may confound BOLD signal and functional connectivity outcomes, especially within the state‐dependent frontostriatal network. Although vigilance‐state differences can be addressed by including a sleep/wake covariate, prior studies show that data collected during non‐sedated sleep in young children remain functionally meaningful. For instance, Yates et al. [68] found that infant/child sleep and awake functional connectivity share a common architecture with state‐dependent modulation, implying that the same basic networks are present across states but connectivity strengths differ. Therefore, using sleep‐based rsfMRI is a necessary and valid approach for studying network development in non‐sedated early childhood populations, providing an interpretable window into foundational neural circuitry. We note that the strongest effects of maternal pre‐pregnancy BMI that we observed were apparent for older children in our study, the majority of whom were not napping. Therefore, we do not believe that sleep status was a significant confound of our positive results but cannot rule out that sleeping status in younger participants may have weakened our ability to detect effects in that group.

The cross‐sectional nature of our study precludes causal inferences, underscoring the need for longitudinal studies to elucidate the developmental trajectories of these functional connectivity patterns and their relationship to cognitive and behaviour outcomes. Also, our modest sample may have limited our ability to identify more subtle effects and to thoroughly explore potential age and sex differences.

Limitations notwithstanding, by leveraging rsfMRI data from a unique cohort including children ranging from 2 to 11 years of age, we illuminate for the first time potential sustained or evolving effects of maternal pre‐pregnancy BMI on offspring frontostriatal brain function beyond infancy and into early and middle childhood, a time where children engage more with their environment and cognitive functions become increasingly sophisticated and impactful on behaviour and health [69]. These findings suggest that maternal obesity may influence offspring neurodevelopment, potentially impacting reward processing and executive functions with relevance to eating behaviour and obesity risk. Since our results suggest that such alterations may have their origins in early childhood, they highlight the importance of early intervention strategies, potentially including preconception and prenatal interventions aimed at optimising maternal metabolic health. Larger studies are needed to confirm and extend our findings, including the possible lateralisation of some of the effects, and prospective studies incorporating gold‐standard measures of maternal and child adiposity and metabolic health as well as behavioural and cognitive measures are needed to more fully understand implications and inform future interventions. Such studies could potentially help to identify specific time windows during which maternal pre‐pregnancy BMI exerts the most significant influence on offspring brain development, thus directly informing the timing of targeted interventions.

## Author Contributions

A.I. and S.C. wrote the paper; A.I., L.C., M.M.K.B. and E.J. analysed data; J.B., A.O., R.C.L. and F.B. conducted the research; S.C., S.C.L.D., V.D. and D.K.‐M. obtained funding and supervised the research; S.C., S.C.L.D. and V.D. designed the study. All authors reviewed and approved the manuscript.

## Funding

This work was supported by the National Institutes of Health, R01DK136602, R01DK113286, UH3OD023313 and Bill and Melinda Gates Foundation.

## Ethics Statement

This study was approved by the Johns Hopkins Ethics Committee (ethics approval code: IRB00118376) and conducted in accordance with the Declaration of Helsinki. Informed written consent was obtained from parents or legal guardians of participants.

## Conflicts of Interest

S.C. declares research funding from Eli Lilly. S.C.L.D. has received grant and salary support from Nestec SA, and speaker fees from Wyeth Nutrition and Mead Johnson Nutrition and is currently employed by the Bill & Melinda Gates Foundation.

## Supporting information


**Data S1:** ijpo70101‐sup‐0001‐Supinfo.docx.

## Data Availability

The data that support the findings of this study are available on request from the corresponding author. The data are not publicly available due to privacy or ethical restrictions.
