# Supplementary material for: Associations of Maternal Pre‐Pregnancy BMI With Frontostriatal Connectivity in Young Children
Source: Pediatr Obes. 2026 Mar 29;21(4):e70101. doi: 10.1111/ijpo.70101 (PMC13033914; doi:10.1111/ijpo.70101)
Supplement: Supplementary file 1 — Data S1: ijpo70101‐sup‐0001‐Supinfo.docx. [file IJPO-21-e70101-s001.docx]

**SUPPLEMENTARY MATERIALS**

***Supplementary Figure 1*.** Reference for network distribution in study-specific child template


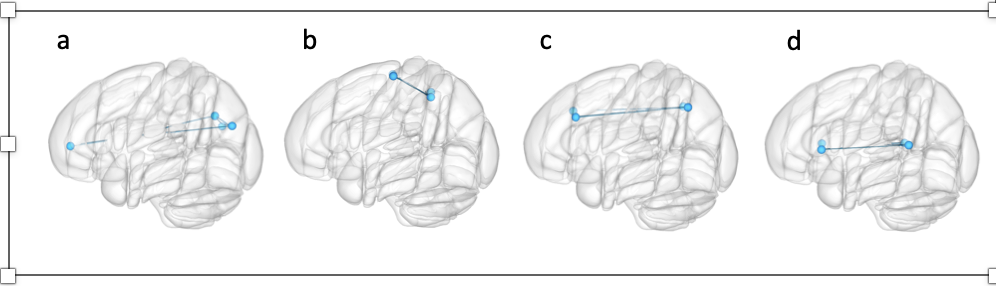

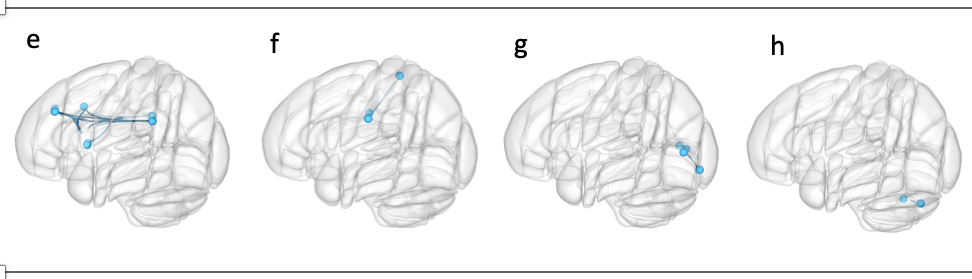


*Footnote: Images depict functional default mode (a), dorsal attention (b), fronto-parietal (c), language (d), salience (e), sensorimotor (f), visual (g), cerebellar (h) networks warped onto an average infant brain.*

***Supplementary Table 1:*** Full regression results across whole sample including effects of covariates

| **Rostral PFC** | | | |  |  |
| --- | --- | --- | --- | --- | --- |
|  |  | Estimate | Std. Error | p | |
| NAcc R – Rostral PFC R | (intercept) | -0.137322 | 0.003745 | 0.200 | |
|  | Pre-pregnancy BMI | 0.004865 | 0.003584 | 0.286 | |
|  | Sex | 0.059600 | 0.050109 | 0.238 | |
|  | Age | 0.011068 | 0.012564 | 0.381 | |
|  | Maternal Education | -0.012289 | 0.027102 | 0.652 | |
|  | Child BMIz | 0.003589 | 0.020421 | 0.861 | |
| NAcc R – Rostral PFC L | (intercept) | -0.129095 | 0.216905 | 0.5537 | |
|  | Pre-pregnancy BMI | 0.009581 | 0.003682 | **0.0119** | |
|  | Sex | 0.050466 | 0.046382 | 0.2804 | |
|  | Age | -0.003262 | 0.011630 | 0.7800 | |
|  | Maternal Education | -0.020653 | 0.025086 | 0.4132 | |
|  | Child BMIz | -0.018192 | 0.018902 | 0.3392 | |
| NAcc L – Rostral PFC R | (intercept) | -3.280e-01 | 2.314e-01 | 0.1610 | |
|  | Pre-pregnancy BMI | 7.890e-03 | 3.810e-02 | **0.0429** | |
|  | Sex | 1.157e-01 | 4.949e-02 | **0.0223** | |
|  | Age | 9.336e-03 | 1.241e-02 | 0.4544 | |
|  | Maternal Edu. | 3.888e-05 | 2.676e-02 | 0.9988 | |
|  | Child BMIz | -1.162e-02 | 2.017e-02 | 0.5662 | |
| NAcc L – Rostral PFC L | (intercept) | -0.020104 | 0.197169 | 0.91909 | |
|  | Pre-pregnancy BMI | 0.008655 | 0.003204 | **0.00921** | |
|  | Sex | 0.129910 | 0.042162 | **0.00297** | |
|  | Age | -0.011490 | 0.010572 | 0.28091 | |
|  | Maternal Education | -0.025114 | 0.022804 | 0.27465 | |
|  | Child BMIz | 0.004822 | 0.017182 | 0.77983 | |
| **Lateral PFC** | | | |  |  |
| NAcc R – Lateral PFC R | (intercept) | -0.193713 | 0.260866 | 0.4603 | |
|  | Pre-pregnancy BMI | 0.005633 | 0.004398 | 0.2057 | |
|  | Sex | 0.079512 | 0.055782 | 0.1586 | |
|  | Age | 0.031130 | 0.013987 | **0.0294** | |
|  | Maternal Edu. | -0.026695 | 0.030171 | 0.3794 | |
|  | Child BMIz | -0.007614 | 0.022733 | 0.7387 | |
| NAcc R – Lateral PFC L | (intercept) | 0.095660 | 0.268274 | 0.7225 | |
|  | Pre-pregnancy BMI | 0.004279 | 0.004351 | 0.3598 | |
|  | Sex | 0.067578 | 0.057367 | 0.2429 | |
|  | Age | 0.011748 | 0.014384 | 0.4169 | |
|  | Maternal Edu. | -0.062382 | 0.031027 | **0.0483** | |
|  | Child BMIz | -0.009407 | 0.023378 | 0.6887 | |
| NAcc L – Lateral PFC R | (intercept) | -0.256261 | 0.225721 | 0.2602 | |
|  | Pre-pregnancy BMI | 0.003434 | 0.003525 | 0.3598 | |
|  | Sex | 0.041860 | 0.048267 | 0.3888 | |
|  | Age | 0.021825 | 0.012103 | 0.0758 . | |
|  | Maternal Education | -0.005533 | 0.026106 | 0.8328 | |
|  | Child BMIz | -0.002800 | 0.019670 | 0.8872 | |
| NAcc L – Lateral PFC L | (intercept) | -0.052035 | 0.214268 | 0.809 | |
|  | Pre-pregnancy BMI | 0.003495 | 0.003327 | 0.298 | |
|  | Sex | 0.068291 | 0.045818 | 0.141 | |
|  | Age | 0.015579 | 0.011488 | 0.180 | |
|  | Maternal Education | -0.033535 | 0.024781 | 0.180 | |
|  | Child BMIz | 0.012053 | 0.018672 | 0.521 | |
| **Frontal pole** | | | |  | |
| NAcc R – Frontal pole R | (intercept) | -0.278767 | 0.241450 | 0.252 | |
|  | PrePregcy_BMI | 0.007537 | 0.003874 | 0.056 | |
|  | Sex | 0.107026 | 0.051630 | **0.042** | |
|  | Age | 0.021538 | 0.012946 | 0.101 | |
|  | Maternal Education | -0.003440 | 0.027925 | 0.902 | |
|  | Child BMIz | -0.012921 | 0.021041 | 0.541 | |
| NAcc R – Frontal pole L | (intercept) | -0.217724 | 0.258335 | 0.4023 | |
|  | Pre-pregnancy BMI | 0.009311 | 0.004452 | **0.0412** | |
|  | Sex | 0.119588 | 0.055241 | **0.0339** | |
|  | Age | 0.010171 | 0.013851 | 0.4653 | |
|  | Maternal Education | -0.025487 | 0.029878 | 0.3966 | |
|  | Child BMIz | -0.027194 | 0.022512 | 0.2312 | |
| NAcc L – Frontal pole R | (intercept) | -0.437117 | 0.210319 | 0.0415 | |
|  | Pre-pregnancy BMI | 0.009689 | 0.003617 | **0.00978** | |
|  | Sex | 0.113814 | 0.044974 | **0.0137** | |
|  | Age | 0.010297 | 0.011277 | 0.3644 | |
|  | Maternal Education | 0.012882 | 0.024325 | 0.5981 | |
|  | Child BMIz | -0.018375 | 0.018328 | 0.3196 | |
| NAcc L – Frontal pole L | (intercept) | -7.230e-02 | 2.213e-01 | 0.74492 | |
|  | Pre-pregnancy BMI | 7.822e-02 | 3.382e-02 | **0.02458** | |
|  | Sex | 1.417e-01 | 4.732e-02 | **0.00383** | |
|  | Age | 7.577e-05 | 1.187e-02 | 0.99492 | |
|  | Maternal Education | -2.885e-02 | 2.560e-02 | 0.26357 | |
|  | Child BMIz | -4.963e-03 | 1.929e-02 | 0.79771 | |

*Footnote: NAcc=nucleus accumbens; PFC=prefrontal cortex; R=right; L=left; Bold indicates significant at p<0.05*

***Supplementary Table 2.*** Regression coefficients for effect of pre-pregnancy maternal BMI on child regional child functional connectivity metrics stratified by maternal pre-pregnancy BMI group.

|  | **Maternal pre-pregnancy BMI group** | | | | | |
| --- | --- | --- | --- | --- | --- | --- |
| **Connectivity with NAcc** | **Healthy-weight (n=47)** | *p* | **Overweight (n=13)** | *p* | **Obesity**  **(n=23)** | *p* |
| **Rostral PFC** |  |  |  |  |  |  |
| NAcc R – Rostral PFC R | 0.0026 (0.0263) | 0.92 | 0.0808 (0.0796) | 0.31 | 0.0825 (0.0642) | 0.20 |
| NAcc R – Rostral PFC L | 0.0207 (0.0222) | 0.36 | 0.1096 (0.0796) | 0.17 | **0.1346 (0.0642)** | **0.04** |
| NAcc L – Rostral PFC R | -0.0123 (0.0252) | 0.62 | 0.1553 (0.0801) | 0.06 | 0.1264 (0.0646) | 0.05 |
| NAcc L – Rostral PFC L | -0.0072 (0.0188) | 0.70 | **0.1903 (0.0660)** | **0.005** | **0.1349 (0.0532)** | **0.01** |
| **Lateral PFC** |  |  |  |  |  |  |
| NAcc R – Lateral PFC R | 0.0044 (0.0293) | 0.88 | 0.0616 (0.0944) | 0.52 | 0.0670 (0.0761) | 0.38 |
| NAcc R – Lateral PFC L | 0.0082 (0.0287) | 0.78 | 0.0486 (0.0934) | 0.60 | 0.0296 (0.0753) | 0.69 |
| NAcc L – Lateral PFC R | -0.0083 (0.0241) | 0.73 | 0.0651 (0.0749) | 0.39 | 0.0624 (0.0604) | 0.30 |
| NAcc L – Lateral PFC L | -0.0001 (0.0012) | 1.00 | 0.0916 (0.0702) | 0.20 | 0.0617 (0.0566) | 0.28 |
| **Frontal pole** |  |  |  |  |  |  |
| NAcc R – Frontal pole R | 0.0037 (0.0279) | 0.89 | 0.0104 (0.0837) | 0.90 | 0.1023 (0.0674) | 0.13 |
| NAcc R – Frontal pole L | 0.0373 (0.0280) | 0.19 | 0.1111 (0.0966) | 0.25 | 0.1037 (0.0779) | 0.19 |
| NAcc L – Frontal pole R | -0.0163 (0.0239) | 0.50 | 0.0621 (0.0766) | 0.42 | **0.1744 (0.0617)** | **0.007** |
| NAcc L – Frontal pole L | 0.0034 (0.0221) | 0.88 | 0.1388 (0.0715) | 0.06 | **0.1218 (0.0576)** | **0.04** |

*Footnote: Models adjust for maternal education and child age, sex and BMIz. Values are β (SE). NAcc=nucleus accumbens; PFC=prefrontal cortex; R=right; L=left. Bold indicates statistical significance at p<0.05.*

***Supplementary Table 3***. Regression coefficients for effect of maternal BMI on child functional connectivity metrics by child sex

| **Connectivity with NAcc** | **Female (n=39)** |  | **Male (n=44)** |  |
| --- | --- | --- | --- | --- |
|  | β (SE) | *p* | β (SE) | *p* |
| **Rostral PFC** |  |  |  |  |
| NAcc R – Rostral PFC R | 0.0089 (0.0243) | 0.71 | 0.0019 (0.0034) | 0.57 |
| NAcc R – Rostral PFC L | 0.0112 (0.0192) | 0.56 | **0.0100 (0.0044)** | **0.03** |
| NAcc L – Rostral PFC R | -0.0167 (0.0258) | 0.52 | **0.0099 (0.0036)** | **0.009** |
| NAcc L – Rostral PFC L | -0.0818 (0.0181) | 0.32 | **0.0109 (0.0039)** | **0.008** |
| **Lateral PFC** |  |  |  |  |
| NAcc R – Lateral PFC R | 0.0175 (0.0266) | 0.51 | 0.0037 (0.0047) | 0.43 |
| NAcc R – Lateral PFC L | 0.0053 (0.0231) | 0.82 | 0.007 (0.0057) | 0.22 |
| NAcc L – Lateral PFC R | 0.0053 (0.0210) | 0.80 | 0.0022 (0.0047) | 0.59 |
| NAcc L – Lateral PFC L | 0.0022 (0.0191) | 0.91 | 0.0019 (0.0043) | 0.64 |
| **Frontal pole** |  |  |  |  |
| NAcc R – Frontal pole R | 0.0276 (0.0260) | 0.29 | **0.0079 (0.0037)** | **0.04** |
| NAcc R – Frontal pole L | 0.0252 (0.0231) | 0.28 | 0.0099 (0.0053) | 0.07 |
| NAcc L – Frontal pole R | -0.0027 (0.0215) | 0.90 | **0.0097 (0.0035)** | **0.009** |
| NAcc L – Frontal pole L | -0.0089 (0.0217) | 0.68 | **0.0083 (0.0038)** | **0.03** |

*Footnote: Models include maternal education and child age, sex and BMIz score.* *NAcc=nucleus accumbens;*

*PFC=prefrontal cortex; R=right; L=left. Bold indicates significant at p<0.05.*

***Supplementary Table 4***. Regression coefficients for effect of maternal BMI on child functional connectivity metrics by age group.

| **Connectivity with NAcc** | **5 years and below (n=43)** |  | **Above 5 years**  **(n=40)** |  |
| --- | --- | --- | --- | --- |
|  | β (SE) | *p* | β (SE) | *p* |
| **Rostral PFC** |  |  |  |  |
| NAcc R – Rostral PFC R | 0.0239 (0.0236) | 0.32 | 0.0046 (0.0037) | 0.23 |
| NAcc R – Rostral PFC L | 0.0105 (0.0187) | 0.57 | **0.0122 (0.0046)** | **0.01** |
| NAcc L – Rostral PFC R | -0.0122 (0.0261) | 0.54 | **0.0106 (0.0040)** | **0.01** |
| NAcc L – Rostral PFC L | -0.0267 (0.0187) | 0.16 | **0.0120 (0.0045)** | **0.01** |
| **Lateral PFC** |  |  |  |  |
| NAcc R – Lateral PFC R | 0.0217 (0.0261) | 0.41 | 0.0041 (0.0051) | 0.43 |
| NAcc R – Lateral PFC L | 0.0144 (0.0227) | 0.53 | 0.0081 (0.0057) | 0.17 |
| NAcc L – Lateral PFC R | 0.0185 (0.0211) | 0.39 | 0.0051 (0.0045) | 0.27 |
| NAcc L – Lateral PFC L | 0.0169 (0.0193) | 0.38 | 0.0023 (0.0048) | 0.40 |
| **Frontal pole** |  |  |  |  |
| NAcc R – Frontal pole R | 0.03113 (0.0262) | 0.24 | 0.0065 (0.0042) | 0.13 |
| NAcc R – Frontal pole L | 0.03368 (0.0239) | 0.16 | 0.0080 (0.0059) | 0.19 |
| NAcc L – Frontal pole R | -0.0045 (0.0221) | 0.84 | **0.0100 (0.0044)** | **0.03** |
| NAcc L – Frontal pole L | -0.0032 (0.0225) | 0.89 | 0.0064 (0.0049) | 0.20 |

*Footnote: Models include maternal education and child age, sex and BMIz score. NAcc=nucleus accumbens;*

*PFC=prefrontal cortex; R=right; L=left. Bold indicates significant at p<0.05.*

***Supplementary Table 5***. Relationships of child resting state functional connectivity metrics with potential confounding variables across all participants.

| **Connectivity with NAcc** | **Maternal education** | **Child age** | **Child sex^♦^** | | | **Child BMIz^a^** |
| --- | --- | --- | --- | --- | --- | --- |
|  | Pearson r (*p*) | Pearson r (*p*) | t-test; Mean±SD | | *t-test(p)* | Pearson r (*p*) |
|  |  |  | Female | Male |  |  |
| **Rostral PFC** |  |  |  |  |  |  |
| NAcc R – Rostral PFC R | -0.082 (0.473) | 0.013 (0.907) | 0.011±0.173 | 0.053±0.219 | -0.960(0.339) | -0.004 (0.977) |
| NAcc R – Rostral PFC L | -0.039 (0.729) | -0.154 (0.163) | -0.144±0.231 | 0.062±0.231 | -1.750(0.083) | 0.182 (0.159) |
| NAcc L – Rostral PFC R | -0.012 (0.917) | -0.032 (0.774) | -0.013±0.161 | 0.087±0.235 | **-2.237(0.028)** | -0.051 (0.696) |
| NAcc L – Rostral PFC L | -0.027(0.811) | 0.0867 (0.435) | 0.001±0.122 | 0.115±0.209 | **-2.998(0.004)** | 0.071 (0.584) |
| **Lateral PFC** |  |  |  |  |  |  |
| NAcc R – Lateral PFC R | -0.097 (0.396) | 0.076 (0.493) | 0.001±0.223 | 0.091±0.229 | -1.816(0.073) | 0.131 (0.315) |
| NAcc R – Lateral PFC L | -0.201 (0.075**)** | -0.086 (0.437) | -0.014±0.216 | 0.058±0.248 | -1.405(0.164) | 0.186 (0.152) |
| NAcc L – Lateral PFC R | 0.002 (0.988) | -0.024 (0.829) | -0.034±0.187 | 0.021±0.199 | -1295(0.199) | **0.253 (0.049)** |
| NAcc L – Lateral PFC L | -0.121 (0.288) | 0.023 (0.837) | 0.002±0.172 | 0.060±0.199 | -1.421(0.159) | **0.276 (0.031)** |
| **Frontal pole** |  |  |  |  |  |  |
| NAcc R – Frontal pole R | 0.006 (0.954) | 0.214 (0.052) | 0.010±0.184 | 0.152±0.222 | **-2.676(0.009)** | -0.009 (0.943) |
| NAcc R – Frontal pole L | -0.026 (0.819) | 0.018 (0.869) | -0.017±0.222 | 0.120±0.233 | **-2.753(0.007)** | 0.186 (0.150) |
| NAcc L – Frontal pole R | 0.108 (0.345) | 0.151 (0.172) | -0.033±0.159 | 0.101±0.199 | **-3.373(0.001)** | 0.060 (0.644) |
| NAcc L – Frontal pole L | -0.000 (0.999) | **0.255 (0.019)*** | -0.005±0.189 | 0.113±0.198 | **-3.365(0.001)** | 0.233 (0.070) |

NAcc=nucleus accumbens; PFC= prefrontal cortex; R=right; L=left;**^♦^**Differences by child sex were tested using independent two-tailed t-test. a = 11 missing; *Bold indicates statistical significance at p<0.05.*
